# Critical Success Factors of Medical Tourism: The Case of South Korea

**DOI:** 10.3390/ijerph16244964

**Published:** 2019-12-06

**Authors:** Soojung Kim, Charles Arcodia, Insin Kim

**Affiliations:** 1 Department of Tourism and Convention, Pusan National University, Pusan 46241, Korea; sj.kim@pusan.ac.kr; 2 Department of Tourism, Sport and Hotel Management, Griffith University, 170 Kessels Rd, Nathan, QLD 4111, Australia; c.arcodia@griffith.edu.au

**Keywords:** Medical tourism, Success factors, Supplier perspectives, South Korea

## Abstract

The purpose of this study was to identify the key success factors of medical tourism using the case of South Korea. Medical tourism refers to the phenomenon of travelling across national borders intentionally to access a variety of medical treatments, especially modern medical treatment. Through conducting semi-structure face-to-face in-depth interviews with the service suppliers of Korean medical tourism, it was discovered that Korean medical tourism has been facilitated by the effect of Hallyu and advanced Korean brand power. More importantly, tourism activities for companions and extra support for patients’ convenience are identified as important success factors of Korean medical tourism, suggesting that the medical tourism industry not only includes medical services but also involves tourism perspectives, supporting the patient and their companions to stay in a comfortable and pleasurable environment. This study generated results which are valuable for both academic and industry perspectives, as this is a field which has not been extensively researched. Medical tourism representatives in other countries can consult these findings to develop the industry.

## 1. Introduction

The medical tourism industry has expanded extensively since the late 1990s [1,2]. Medical tourism generally refers to travelling across international borders to obtain a range of medical services [3,4,5]. Patients looking for affordable, as well as high quality medical care travel to medical centers in other countries. The majority of medical tourists who travel from developed countries to developing countries are motivated by the out-of-pocket cost of healthcare, long waiting times to access the service or to access specific procedures which are not available in their home countries [6,7,8,9,10,11]. The international medical tourism market size is expected to reach USD 131.35 billion by 2025, with an average annual rate of 20% [12].

Although there are a variety of destinations around the world, Asia is considered as a major medical tourism destination [13,14,15,16]. Asian countries, including Thailand, India, Malaysia, Singapore and South Korea, have enthusiastically promoted such services, and the competition to attract more international medical tourists has become more intense. The rise of the medical tourism industry in Asia can be partly attributed to the Asian financial crisis [14,17]. The Asian financial crisis discouraged much of middle-class Asia to pay for private healthcare, hence private hospitals have expanded by targeting international patients in order to generate revenue. Moreover, the medical tourism industry brings to these destinations benefits such as increasing gross domestic product (GDP), improving medical services and generating foreign exchange [3].

Although the lucrative medical tourism industry has captured the attention of Asian countries, there is a paucity of literature which focuses on medical tourism [3] and even fewer studies have explored the industry from supply perspectives [17]. Investigating the service suppliers’ perspective, however, is significant because it is the suppliers’ task to transform resources and demand requirements into value [18]. The creation of customer value is essential in sustaining a competitive advantage in the growing medical tourism industry [19,20] as it leads to affirmative customer evaluations [21]. Therefore, this study aims to identify and analyze the critical success factors of medical tourism from the service providers’ perspectives. This study adopted the case study approach, with South Korea as a representative case, and conducted in-depth interviews providing deeper and comprehensive understandings of this social phenomenon.

## 2. Definition of Medical Tourism

Although the medical tourism industry has expanded rapidly around the world, one of the major problems is that there is no consistent definition of medical tourism in the literature [5,6,14,22,23,24,25]. Connell [14] suggested some reasons for the difficulties in defining the term medical tourism; the various medical procedures provided, the variety of medical tourists’ motivations to access the services, the diverse socio-economic levels of medical tourists, the lack of features of tourism and leisure in medical tourism and the amount of resources allocated to particular activities. Some authors even criticized defining the term medical tourism [25,26], by arguing that the term medical tourism evokes fun, relaxation and pleasure images rather than serious health treatments. It is, however, more useful and valuable to have a consistent definition of medical tourism in circumstances where the number of medical tourists and the amount of revenue from the industry has increased around the world [14,17,26].

There are many terms which are used to describe the phenomenon of travelling to other countries to access healthcare; for example, medical tourism, health tourism, wellness tourism, transplant tourism, reproductive tourism and dental tourism [8,14,25,27]. Although some authors [28,29,30] used medical tourism and health tourism interchangeably, or considered medical tourism as one of the subcategories of health tourism [8], almost all authors distinguished medical tourism from health tourism [14,17,26,27,31,32,33]. Connell [17] distinguished health tourism and medical tourism by limiting medical tourism to “people travel often long distances to overseas countries to obtain medical, dental and surgical care while simultaneously being holidaymakers, in a more conventional sense,” (p. 1094), compared to the much broader concept of health tourism which indicates travelling with the main purpose of beneficial health outcomes. Carrera and Bridges [34] differentiated medical tourism from health tourism by emphasizing medical intervention in medical tourism products such as dentistry, cardiac surgery or cosmetic surgery in the former, and, non-medical intervention in health tourism products, such as enjoying a spa, visiting hot springs or receiving massages in the latter. Hall [27] distinguished between wellness, health and medical tourism based on medical tourists’ health statuses and travel purposes; for example, wellness tourism occurs when people, who are enjoying their well-being, travel with a health promotive purpose, and health tourism is when healthy people travel to access medical treatment with an illness preventive purpose; on the other hand, medical tourism is when people who have an illness travel to have medical treatment with an illness-curative purpose.

While it is difficult to define the term medical tourism, there are some common characteristics among the medical tourism definitions. The first characteristic of medical tourism is that medical tourists travel abroad for healthcare based on their own intention, which means that medical tourism is elective and discretionary, unlike formal cross-border institutional transfers [7,14,35]. There is no intention in a patient who is transferred from their home country to another country’s healthcare system by a physician’s decision. Unlike this patient, medical tourists decide to participate in medical tourism by themselves intentionally. Johnston, Crooks, Adams, Snyder and Kingsbury [35] also emphasized medical tourists’ intentions when defining medical tourism as “Medical tourism occurs when patients intentionally leave their home countries for non-emergency medical care that is not part of a cross-border care arrangement” (p. 1). Another feature of medical tourism is that the services tend to have medical intervention, contrary to other forms of health-related tourism products [8,13,14,22,34]. While health-tourism travelers tend to seek a spa or a massage, which do not have medical intervention, medical tourists pursue access to medical intervention treatment such as cardiac surgery or cosmetic surgery. Lastly, the term medical tourism is applied to the tourists who are not moving within a country but travelling across national borders to access medical services [6,14,25,36]. There are also movements within nations to access medical treatment; however, the term medical tourists refers to patients who cross national borders for medical procedures in almost all the literature.

Based on these characteristics, medical tourism in this study is defined as the phenomenon of travelling across national borders to intentionally access a variety of medical treatments which can be necessary or elective for medical tourists. These medical treatments can be all services which are offered in hospitals, including serious invasive surgeries such as cardiac surgery, but also light procedures such as health screenings as well as Botox or derma fillers. Activities such as visiting Korea to enjoy spas or massages which do not occur in hospitals are not considered as medical tourism in this study.

## 3. Medical Tourism Success Factors

### 3.1. Increased Demand

There is no doubt about the increasing market size of medical tourism and the increasing demand for services [14,27,35,37,38,39,40]. The reasons for the increasing number of medical tourists travelling from developed to developing countries can be economically explained by Oyewole [41]. This author explained that increasing disposable income in developed countries facilitates the purchasing of services in developing countries where the pricing is very competitive. In particular, the US is expected to provide a huge medical-tourism market because of the US’s expensive treatment costs, long waiting times to access medical services, serious health insurance problems, and out-of-pocket expenses for elective surgery, even for people with health insurance [13,25,42,43,44]. Turner [25] explained that the reasons for the serious health insurance problems in the U.S. are the expensive health insurance fees and the high procedure costs. Expensive health insurance fees mean millions of Americans are underinsured on low-budget plans that cover only a fraction of the healthcare costs and services [45]. Even people with insurance cannot afford many medical procedures because of high deductibles and co-payments. For these reasons, all communities, from individuals to state governments, in the US are interested in medical tourism, which can help solve such problems [38].

Furthermore, most patients in Canada and the UK are exposed to a lack of timely access to elective procedures, although both countries provide universal healthcare coverage. This lack of timeliness motivates residents to participate in medical tourism [25,42]. However, it is not just inconvenient healthcare systems in developed countries that are leading to the growth of the medical tourism industry; other factors are also important, such as the serious growth in aging populations [46,47], the development of the Internet, the affordability of overseas transportation [48], improved standards in international healthcare and the World Trade Organization’s (WTO) multilateral trade agreement such as the General Agreement on Trade in Services (GATS), including health services [25,37,43,49,50].

### 3.2. Suppliers’ Rigorous Investment in the Industry

Increasing demand for medical tourism has provoked fierce competition among several countries which enthusiastically promote themselves as medical tourism destinations [14,31,42]. At the present time, at least 28 countries are competing worldwide for the medical tourism business [51]. These countries can be divided based on continents: South Africa in Africa, Thailand, Singapore, Malaysia, South Korea, Dubai, India, Israel, Jordan and the United Arab Emirates in Asia, the Czech Republic, Hungary and Poland in Europe, and Argentina, Brazil, Costa Rica, Mexico and Panama in Latin America [38,52]. Awadzi and Panda [42] divided these destinations into countries actively promoting medical tourism, such as Greece, South Africa, India, Malaysia, Philippines, Thailand and Singapore, and countries relatively new to the field, including China, Germany, Canada, Costa Rica, Hungary, Dubai, Argentina and Cuba.

Although there are a variety of medical tourism destinations around the world, Asia is considered a major destination [6,13,15,30,43,53]. As mentioned earlier, the rise of the medical tourism industry in Asia can be partially attributable to the Asian financial crisis [14,17]. The Asian financial crisis discouraged much of middle-class Asia to pay for private healthcare; consequently, private hospitals focused their attention on international patients in order to generate revenue. Malaysia and Thailand were the leaders in the medical tourism industry, followed by other south-east Asian countries and India.

The main barriers to growth in the sector in Thailand, Malaysia and India are the negative perceptions toward healthcare in developing countries and a stereotypical perception of low cost equating to low quality [16,26,29,36,54]. In order to overcome these challenges, hospitals and healthcare institutions in major destinations have tried to receive international accreditation to prove the quality of their systems [31,55]. Joint Commission International (JCI), which is given by the most popular healthcare accreditation group Joint Commission on Accreditation of Healthcare Organizations (JCAHO), is the most desired and common accreditation for international medical tourism service providers [8,25]. The international accreditation increases the credibility of healthcare services in hospitals, which can be a competitive marketing tool for attracting international visitors.

### 3.3. The Role of the Medical Tourism Agency

Medical tourism agencies, also known as medical tourism facilitators, are the organizations which specialize in arranging suitable foreign hospitals and treatment, transportation and lodging during recuperation [43]. A few medical tourism agencies exist with branches both in the departure and destination countries, but most of the agencies are not of a huge size and have a short history [25,52]. The medical tourism agencies’ websites provide information which is useful to medical tourists, such as accredited hospitals, affordable prices, exceptional experiences, time advantages, high quality and reliable care [52]. Even though medical tourism agencies are identified as one of the service providers in the medical tourism industry, they tend not to be obliged to receive accreditation for their duty. Turner [38] advised that it is important that medical tourism agencies and facilitators should be subject to receiving external evaluation and accreditations which are based on meeting the criteria of transparent, appropriate, and well-defined standards of practice, as well as conforming to legal and ethical issues such as privacy of patient information and medical records and fair advertising.

## 4. Medical Tourism in South Korea

South Korea is emerging as a new medical tourism destination but ranks sixth among nine major Asian destinations; Thailand, Singapore, India, Malaysia, the Philippines, Korea, Jordan, the United Arab Emirates, and Israel [15,50]. South Korea is rapidly emerging as one of the medical tourism destinations with potential growth powers of advanced medical treatment technology and a reputation for plastic surgery [49,56,57,58]. In 2009, Korean medical tourism provided services for around 60,000 medical tourists and in 2017 the total number of international patients was 320,000. The country is forecasted to attract one million medical tourists by 2020 [58]. As demand for services in Korea increases, there is also an increasing number of service suppliers. A total of 31 medical tourism healthcare providers have been certified by Joint Commission International in Korea.

The main nationalities who visit Korea to access medical treatment are those from China, the U.S., Japan, Russia and Mongolia. The Japanese are interested in aesthetic treatments and crucial health treatments; on the other hand, the Chinese display a high demand for simple treatments, aesthetic and healthcare services [15]. Japan and China are selected as the major target countries in Korean medical tourism, and a few studies investigate consumer behaviors regarding them. Asian perceptions of beauty and the broader context of Korean popular culture encourage Korea to become a plastic surgery destination for a few East and South-East Asian countries [14,15,57]. Moreover, the numbers of patients who are from China, Russia, Vietnam, Saudi Arabia, Kazakhstan, Uzbekistan and Cambodia have dramatically increased, with an over 100 per cent average annual increase rate [59]. Internal medicine and healthcare screenings are the top two departments the medical tourists are mainly looking for; on the other hand, there is a radical increase in the number of medical tourists visiting Korea to access plastic surgery, traditional Korean medicine and ophthalmology [59].

The major organizations promoting Korean medical tourism the most actively are the medical centers, the central government and the municipal governments which are the Ministry of Health and Welfare and the Ministry of Culture, Sports and Tourism [60]. The Ministry of Health and Welfare aims to improve the overall population health and provide equity of opportunity to social participation, and facilitates medical tourism as a part of strategic global cooperation [61]. The Ministry of Culture, Sports and Tourism aims to build a sustainable society and regards medical tourism as a major facilitator to achieve an economically, socially and culturally sustainable society [62]. The government has considered medical tourism to be the new growth industry and actively participates in expanding the industry by implementing overseas marketing, fostering human resources, expanding one stop service centers and establishing The Korean International Medical Association (KIMA), which comprises of private medical facilities and governmental organizations [50,60].

In spite of Korea’s popularity as a medical tourism destination, there is a paucity of literature which studies medical tourism in Korea, because Korea has only recently entered the medical tourism market compared to Thailand and Malaysia [15,16,54,56,63]. Among the few reported studies, some focused on analyzing the demand perspectives, such as how cultural differences affect the choice of destination [15,40] and the formation of Japanese tourists’ travel plans for medical treatment in Korea [56]. One article investigated the key developmental characteristics of medical tourism in Korea [39]. As the medical tourism industry becomes more competitive, identifying demand expectations and requirements provides key resources for establishing a competitive advantage; however, investigating the service suppliers’ perspective is also important because it is the suppliers’ tasks that transform resources and demand requirements into value and a competitive advantage [18]. Therefore, the main purpose of this study is to identify and analyze the success factors in Korean medical tourism from the service provider perspective.

## 5. Methodology

### 5.1. Case Study

This paper reports on a case study of South Korea as a single representative study. A case study uses an empirical approach and a particular person, group or situation to investigate a contemporary phenomenon [64], such as medical tourism. A case study approach is useful in the exploratory stage of the investigation, as it enables the researcher to obtain a conceptual insight into events through the interpretation and combining of theory with the events [65,66]. Korean medical tourism has developed in earnest since 2009, when the government selected the healthcare industry as one of the new engines for economic growth, and requires further investigation [67] to maintain its popularity and competitiveness over other medical tourism destinations.

### 5.2. Samples of this Study

**Figure 1:**
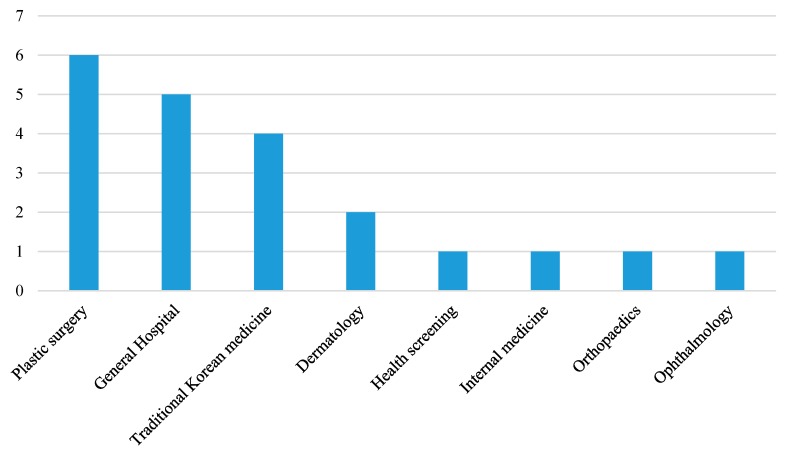
Components of the hospital.

Purposive sampling was used to determine the medical tourism suppliers to be included in this study. The sample organizations were consciously selected based on their expertise and experience [68], including their experience in promoting medical services, attracting international medical tourists and providing medical tourism services. Participants were comprised of 21 service providers from hospitals, five from medical tourism facilitators and two from government organizations. More specifically, various departments of hospitals participated in the in-depth interviews, which included general hospitals offering diverse specialist services, specialist plastic surgery hospitals, and other service providers offering Korean traditional medicine, dermatology, health screening, internal medicine, orthopedics and ophthalmology, as Figure 1 describes.

### 5.3. Data Collection and Analysis

This study conducted semi-structured face-to-face in-depth interviews with service suppliers of Korean medical tourism to obtain richer and more meaningful opinions toward the key success factors of Korean medical tourism. The in-depth interviews were designed to take approximately 30 min and they consisted of open-ended questions which provided the important concerns of participants and provided a rich source of multiple insights for further investigation [69]. The collected data were analyzed by using content analysis, which is commonly used in social science research to analyze textual data such as interview transcriptions or travel diaries [70]. In this study, the collected data were analyzed by using quantitative content analysis which counted text populations within categories, which is a clear and reasonable methodology based on the assumption that the most frequent theme is the most important [71,72]. The categories were inductively extracted from the collected data. As many headings as were highlighted through reading the interview transcripts and indefinite categories were created.

## 6. Results and Discussion

**Figure 2:**
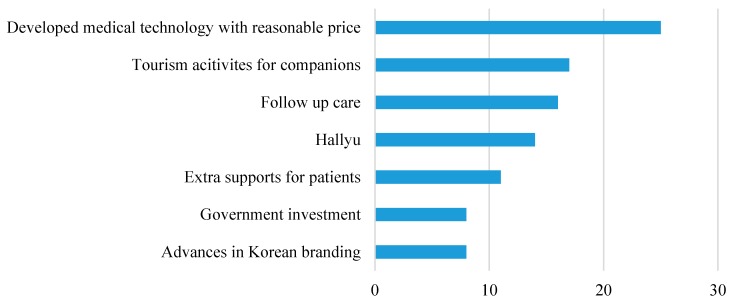
The number of respondents indicating each success factor.

All of the interviewees recognized the increased number of medical tourists in Korea, and the key factors identified for attracting more medical tourists to South Korea included the developed medical technology with a reasonable price, tourism activities for companions, follow-up care, the Hallyu effect, extra supports for patients, strong government investment in medical tourism, and advances in the Korean branding. The full list of identified factors is itemized in Figure 2.

### 6.1. Developed Medical Technology with a Reasonable Price

Medical tourism suppliers regarded high levels of medical technology as the strongest success factor followed by reasonable prices for the participants, though reasonable pricing is considered as the first motivation for medical tourists to go abroad to access medical treatment. This is similar to the conclusions provided by Crooks, Kingsbury, Snyder and Johnston [6], which suggests that high levels of medical technology is more emphasized than low cost in marketing materials because emphasizing low cost can increase the suspicion of low cost being linked with low quality.

### 6.2. Tourism Activities for Companions

The service providers argue that tourism activities such as shopping, sightseeing and/or casinos provide competitiveness for Korean medical tourism. Shopping, among other activities, is developed in Korea, providing greater product variety within brands at lower prices than in China or Japan [73], the major sources of medical tourists and patients [50]. Participant 1 explained that “Chinese and Japanese enjoy shopping and they said that there are more various products with lower price in Korea even though same brand”, while Participant 15 additionally mentioned that “Plastic surgery medical tourists tend to go shopping rather than go sightseeing. They are likely to purchase luxurious products, which are cheaper than in their home countries, even purchasing Korean products, which is also an aspect of the Hallyu”. According to Lam, Du Cros and Vong [24] Chinese potential medical tourists prefer shopping and sightseeing, while the least preferred activity is gambling. Chinese medical tourists’ preference for shopping is another reason why Chinese medical tourists occupied the largest market in Korean medical tourism in 2012, as Korean shopping is well developed.

In spite of shopping being selected as a major tourism activity which medical tourists can enjoy, three interview participants pointed out the lack of infrastructure which medical tourists can enjoy for recuperative purposes, as Participant 6 argues that “In fact, Korea does not have enough holiday infrastructures which medical tourists can enjoy during the post-care period. Medical tourists visit Korea with the purpose of improving health and relaxation. However, even though Korea has well-developed shopping areas, there is a lack of places for relaxation and recuperation, such as spas or massages.”

In Thailand, the spa and holiday services are well established and have been internationally and domestically promoted [74]. Further, Bumrungrab hospital, a major hospital treating medical tourists in Thailand, has yoga facilities which medical tourists or their companions can enjoy. Wang [75] also recommended that medical tourism destinations establish healthcare services and programs which provide memorable and relaxing holidays. The primary purpose of participating in medical tourism is health improvement and thus the service providers need to offer extra activities which medical tourists can enjoy to improve their health; Participant 23 strongly suggested that “We need to establish an infrastructure which provides various ranges of health care programs such as spa, fitness center and meditation centers.”

### 6.3. Follow up Care

Follow-up care was identified as the major success factor by 18 out of the 28 respondents. Follow-up care is offered in two ways: online or in the local hospitals. Online follow-up care includes administration by email, Skype or telephone, and six medical tourism health providers have partnerships with local hospitals. Concerns over a lack of continuous care is one of the challenges of medical tourism, because medical tourists normally return to their home countries after one or two weeks of a brief recovery period is complete [6,30,50]. If unexpected side effects or results arise when they are in their home countries, it is difficult for medical tourists to access immediate medical attention, and it requires a lot of physical, mental, and financial commitment and where-with-all to visit the doctor who performed the surgery [30]. In order to overcome these challenges, a few Korean hospitals have partnerships with home-country hospitals, which help medical tourists to more readily access an immediate diagnosis of unexpected results or side effects. Some Korean physicians, especially plastic surgery specialists, go to these home-county partnership hospitals on weekends to provide support medical services. According to Davies and Han [76], not only do Korean plastic surgeons go to Chinese-owned clinics to provide medical services, but from 2005 onwards, they also own and operate clinics in Shanghai and Beijing. For example, Participant 22 mentioned that:

“We have a partnership with a hospital in Japan, which operates franchisees in Japan. In China, we opened a branch in last year, 2012. Chinese specialists had trained in the main hospital in Korea, and there are also Korean specialists in China. As opening a plastic surgery branch in China, Chinese medical tourists who are required follow up care can receive it in their home countries more comfortably. Moreover, medical tourists can receive Korean medical surgeries in their home countries because there are Chinese specialists who trained in the main hospital and Korean specialists as well.”

Not every interviewee, however, showed positive attitudes towards having partnerships with the local hospitals. For example, Participant 18 mentioned that:

“Medical tourists are not from one region in countries. Even though we have a partnership with a hospital in east China, it is too far for medical tourists in west China to go for follow-up care. Therefore, we have contact with medical tourists by online or telephone and if they need, we advise them to go the nearest hospital in their home town. With these reasons, we give detailed specialists’ notes to medical tourists when they leave for their home countries.”

The sizes of China and Russia make it financially and logically prohibitive to form partnerships with local hospitals in all districts, especially as medical tourists could come from anywhere in these countries to Korea. Therefore, even though Korean hospitals have partnerships with a few hospitals in China and Russia, not every medical tourist can go to these partnership hospitals. Johnston, Crooks and Snyder [7] also presented a similar finding, that Canadian medical tourists are likely to go to family doctors for some degree of diagnosis when they return to their home countries.

The importance of follow-up care was demonstrated by Pickert [77], who stated that the American Medical Association introduced a set of guidelines for medical tourism that included proper follow-up care, and thus Korean medical tourism needs to establish more systematic follow-up programs to reduce post-treatment concerns which dissuade medical tourists from leaving their home-county to access medical treatment.

### 6.4. The Effect of Hallyu

Hallyu, which is also referred to by some as the Korean Wave, refers to “the phenomenon of Korean pop culture, such as TV dramas, films, pop music, fashion and online games being widely embraced and shared among the people of Japan, China, Hong Kong, Taiwan and other Asian countries” [78] (p.115). The word Hallyu was first mentioned by the Chinese mass media in 2002, but Hallyu was enthusiastically embraced from 2003 when a Korean drama named ‘Winter Sonata’ was first shown in Japan [56]. Beginning with the success of ‘Winter Sonata’, more Korean dramas have been marketed directly to other Asian countries. Hallyu has directly increased sales from Korean drama exports and has indirectly positively influenced Korean tourism [78,79,80,81].

The huge effect of Hallyu is not limited to the mainstream Korean tourism industry because 14 responses mentioned Hallyu as the major reason for the increasing numbers of medical tourists in Korea. Hallyu has influenced Korean medical tourism in two specific ways; the awareness of and familiarity with Korea has increased, and surgically beautified celebrities have endorsed Korean plastic surgery. Firstly, as Hallyu has spread to other Asian countries, the awareness of Korean culture has increased and people feel a closer familiarity to Korea [82,83]. Since familiarity with destinations is a facilitator for potential medical tourists to select a country [14], a closer familiarity and the increased awareness of Korea through Hallyu positively affects Korean medical tourism [67]. Moreover, the representatives from plastic surgery hospitals explained that some young or middle-aged women who enjoy watching Korean TV programs and films have the desire to look like Korean celebrities. Furthermore, receiving plastic surgery is not a hidden fact anymore in Korea. It is common to present surgically modified celebrities in testament to particular hospitals [76]. In other words, Korean celebrities help endorse plastic surgery, which attracts potential medical tourists to participate in Korean medical tourism, as Participant 1 explains:

“Hallyu standardized the criteria of beauty, which means that people in other countries (Asia) also have similar views about beauty. Potential medical tourists, who yearn to look like Korean celebrities, have intention to access plastic surgery in Korea where the celebrities live. Moreover, plastic surgery has become common in Korea, and people do not hush up the fact that they received the surgeries. I think this trend has spread to other countries through Hallyu, and thus people in other countries have been more interested in Korean plastic surgery.”

This interesting result of Hallyu as a phenomenon is recognized in a number of Asian countries, encouraging participation in Korean plastic surgery, especially from China. As income has risen in China, interest in beauty has also increased [84]. Ford [84] further explained the reasons for the popularity of Korean plastic surgery in China, as there being more experienced specialists and more developed techniques in Korea than in China. Moreover, since most Asians have similar cosmetic goals, Korean specialists and Chinese medical tourists find it easier to communicate [84]. Participant 18 also stated the reasons for the popularity of Korean plastic surgery in China were geographical closeness and the positive changes in Chinese perceptions toward plastic surgery, mentioning that “Hallyu occurs in various Asian countries. However, the Chinese especially visit Korea for plastic surgery because China is near Korea, as well as I think Chinese perceptions toward plastic surgery have changed positively.”

### 6.5. Extra Support for Patients

Korean medical tourism service providers have provided extra services including lower prices for accommodation and pick-up services as well as introducing sightseeing possibilities. Two hospitals provide Russian broadcasts for Russian patients, and another two hospitals provide companion meals together when offering the patients’ meals. The importance of extra support for the patients’ convenience supports the conclusion by Han and Hyun [85], arguing the critical role of medical and service quality in customers satisfaction.

Wang [75] recommended that medical centers link with hotels or tourism agencies to offer all-inclusive holiday packages including pick-up services, accommodation, shopping and/or sightseeing. Korean medical tourism has partly developed partnerships between hospitals and hotels [86,87]; however, there is a lack of partnership with tourism agencies, which would be necessary to provide all-inclusive holiday packages. The service providers also have recognized a lack of partnerships with tourism agencies, as Participant 24 mentioned that “Mostly, hospitals do airport pick up services because there is a lack of medical tourism agencies which treat medical tourists systemically.”

### 6.6. Government Investment

The Korean government’s active involvement and investment in the medical tourism industry was selected as another major factor in attracting medical tourists. The Korean government determined medical tourism as a new growth engine industry in 2009 and has actively invested in the industry by regulating policies supporting the industry and promoting high medical services abroad. For example, Participant 21 strongly argued that “The primary factor increasing the number of medical tourists is the government’s investments in the industry. The government has invested and actively promoted Korean medical tourism since 2009 and is calling the industry a new growth engine industry”.

The importance of the government’s participation in developing medical tourism has been strongly identified in other destinations as well, such as Malaysia, Croatia, Hong Kong, Singapore and Thailand [4,6,32,36]. The governments in the major medical tourism destinations regard the medical tourism trade as an important resource for economic and social development and, similarly, Korea has identified the medical tourism industry as the new growth engine industry. In order to reduce entry barriers for medical tourists and increase medical tourists’ inflow, the Korean government introduced medical visas similar to the ones introduced by other major medical tourism destinations such as Thailand, Singapore and Malaysia [22]. Compared to the Korean government, governments in other major destinations have provided more active financial support. The Malaysian government has provided tax incentives or material incentives for setting up international patient units and for expenses incurred in receiving international accreditation such as JCI [49]. When Singapore planned to establish itself as a ‘Biomedical Hub’ in the 1990s, the government offered various incentives to the biomedical industry, such as low corporate tax rates or low personal income tax rates, which helped develop medical tourism [25]. Skountridaki [88] explained that the reasons why these countries consider the industry as an important sector in terms of enabling the diversification of their economies are that it attracts foreign investment, promotes job creation, builds the health services industry, and prevents the outflow of healthcare providers to wealthier nations.

### 6.7. Advances in Korea Branding

Moreover, advanced Korean brand power is also attracting medical tourists; as Participant 11 mentioned, “Korea has experienced advanced international position caused by Hallyu as well as major Korean companies such as Samsung and LG. These various factors positively shape the image toward Korea for potential medical tourists”. This advanced brand power includes a higher international position generally in the IT and mobile phone industry, as well as establishing a stronger brand name for plastic surgery in the medical tourism industry. Interbrand, which is the world’s leading brand consultancy, announced that Samsung entered the global top ten brands by taking the sixth position, and Hyundai and Kia entered the global top 100 brands category [89]. Lee and Jang [60] suggested that the success of Samsung’s Galaxy series smart phone and TV attributed to the growth in Samsung’s brand value. This advanced brand power has shaped the image of Korea and this can positively impact potential medical tourists to select the destination [90,91] because the major destinations, of which most are developing countries, have suffered from negative perceptions about health services in these countries. Social and cultural compatibility is also an important factor in destination selection for medical tourists [35,43].

Korean medical tourism has established a strong brand name for plastic surgery. Davies and Han [76] also mentioned that Korean plastic surgery is a superior brand in China, and Lee [92] highlighted the popularity of plastic surgery in Korean medical tourism. Participant 10 described this, saying “Korea has established brand identity for plastic surgery, which makes people believe that receiving plastic surgery in Korea will be successful”. According to Lee [92], Korea has the world’s highest rate of plastic surgery, and this may produce high plastic surgery skills in Korea. Korean medical tourism provides various medical services, such as traditional Korean medicine, health screening, internal medicine, orthopedics, ophthalmology and dentistry. The reason, however, why Korean medical tourism has specialized in plastic surgery can be explained by the effects of Hallyu and high plastic surgery skills.

According to Cook [31], major medical tourism destinations have invested time and money in establishing a brand name which is similar to the reputation of products; for example, wellness tourism and gender reassignment surgery in Thailand or orthopedic and cardiac surgery in India. Establishing a reputation for plastic surgery in Korea can differentiate it from other major destinations in the very competitive medical tourism industry.

## 7. Conclusions

This study explored the key success factors of medical tourism with a case study about Korea. Overall, Korean medical tourism has gained a competitive advantage among other destinations, with the effect of Hallyu and Korean brand power which both generates credibility to Korean medical skills. Hallyu has increased familiarity with Korea and had a positive impact by increasing the number of medical tourists, particularly for plastic surgery. Advanced Korean brand power, such as Samsung, has positively shaped the image of Korea. In addition, Korean medical tourism gains a competitive advantage not only through satisfying medical tourists seeking medical treatment at a reasonable price, but also generates customer values such as providing tourism activities for companions and follow-up care, which is a concern for potential medical tourists mainly concern. In order to reduce medical tourists’ concerns regarding follow-up care, service providers have continuous contact with medical tourists online and through home-county partnership hospitals provide medical support for tourists in their home countries.

While some research on medical tourism investigated the industry from the demand side, such as consumer behaviors [50], this study is distinctive because it investigates Korean medical tourism from the supply side. Moreover, although there are many studies exploring the challenges in medical tourism, such as concerns about follow-up care and cultural differences, there was little research emphasizing the service providers’ efforts to reduce the challenges. The results in this study address a gap in the literature in finding the service providers’ efforts to overcome the identified challenges. Practically, this study generated results which are valuable for the representatives of the medical tourism industry. Medical tourism representatives in the countries where the industry is in the initial stages can consult the findings to develop the industry.

While this study addressed the research gap and provided valuable findings for the medical tourism industry, there are still several limitations. To explore Korean medical tourism from the service providers’ perspectives is one aspect of understanding this phenomenon. It is hoped that to investigate the industry from practical medical tourists’ views or other service providers’ perspectives, such as insurance companies and related accommodation should be useful future research. Moreover, it may be also valuable to focus on a specific group of the service providers or a specific medical department. Moreover, based on the results in this study, comparative analysis between Korean medical tourism and medical tourism in other major countries such as Thailand, India, Singapore or Malaysia can be used for future studies by using online narratives of medical tourists to Korea.
